# Empirical design of population health strategies accounting for the distribution of population health risks

**DOI:** 10.1016/j.ssmph.2024.101741

**Published:** 2024-12-17

**Authors:** Ayumi Hashimoto, Hideki Hashimoto

**Affiliations:** a Graduate School of Medicine, The University of Tokyo, Tokyo, Japan; b Faculty of Economics, Keio University, Tokyo, Japan; c David Geffen School of Medicine, University of California, Los Angeles, CA, USA; d Department of Health and Social Behavior, School of Public Health, The University of Tokyo, Tokyo, Japan

**Keywords:** Distributions, Quantile regression-based decomposition, Population health strategies, Health equity

## Abstract

Recent discussions in epidemiology have emphasised the need to estimate the heterogeneous effects of risk factors across the distribution of health outcomes for better aetiological understanding of the determinants of population health. We propose using quantile regression-based decomposition to expand the empirical discussion on population health intervention strategies for health equity by incorporating population homogeneity/heterogeneity in the risk–outcome association. We theorised that the ‘proportionate universalism’ approach presumes population homogeneity in the risk–outcome association with varying risk intensities, which decomposition analysis shows as the ‘covariates part’ between groups. Conversely, the ‘targeted approach’ assumes population heterogeneity in the risk–outcome association across the outcome range, which the analysis identifies as the ‘coefficients part’. Our demonstration, using a case of education-related disparity in dietary behaviours, exemplified that differences between education groups were mainly explained by the coefficients part. This finding suggests heterogeneity in their risk profiles, necessitating a ‘targeted approach’ across outcome quantiles to close the gap. The ‘proportionate universalism’ strategy could be partially applied to specific quantile segments where the covariates part remained significant as a supplementary intervention. However, simply increasing the magnitude of certain risk factors (e.g., income) showed conflicting directions between covariates and coefficients parts. Structural modifications of risk–outcome associations would therefore be more equitable. We also discuss the potential strengths and limitations of the analysis, suggesting that it may be complemented by data-driven methods using machine learning to identify discriminating risk factors for population health equity.

## Highlights

• Distributional heterogeneity of risk factors shapes populations health patterns.
• Quantile regression decomposition is used to assess distributional heterogeneity.
• We propose a framework for interpreting results in health strategy decisions.
• The framework's usefulness is shown in an education-related dietary example.
• The proposed framework is broadly applicable for health equity planning.

## 1 Introduction

In recent discussions in epidemiology, Bann et al. (Bann et al., 2020) argued that the effect estimation of average differences in outcomes, as examined in dominant epidemiology studies, overlooks the importance of risk factors that are heterogeneously associated across the distribution of targeted outcomes. They demonstrated the potential usefulness of quantile regression models in exploring differential effects across the range of the outcome distribution within the population, thereby deepening the aetiological understanding of the determinants of population health.

In a similar line of argument, Siddiqi et al. (Siddiqi et al., 2018) proposed a ‘consequential’ shift to distributional inequalities of risks and outcomes between populations to address the determinants of population health, following the arguments raised by Rose (Rose, 1985) and Jones (Jones, 1997). To this end, Siddiqi et al. (Siddiqi et al., 2018) suggested using a decomposition technique based on propensity score weighting of subpopulation group affiliation. Although the use of decomposition techniques is not new in epidemiological studies on health inequality, most have relied on Blinder–Oaxaca decomposition based on linear regression estimators (Kino & Kawachi, 2020; Rahimi et al., 2021). The DiNardo–Fortin–Lemieux decomposition adopted by Siddiqi et al. (Siddiqi et al., 2018) extends to non-linear models with the use of semi-parametric models. In both analytic frameworks, however, distributional inequalities in the outcome are decomposed into components explained by between-group differences in the distribution of predictors and those unexplained by known predictors. These methods primarily address the average treatment effect between subpopulation groups.

By contrast, using a quantile regression-based decomposition approach allows us to articulate the heterogeneity in the distribution of predictor risk factors and outcomes between population subgroups across the range of the outcome distribution (Chernozhukov et al., 2013; Machado & Mata, 2005). Some epidemiological studies have already applied this technique to explain the sources of health inequality between social groups (Dunn et al., 2012; O'Donnell et al., 2009). In more detail, it accounts for different distributions of risk factors between comparative groups in each of the outcome quantiles, which are further decomposed into between-group differences in the levels of risks and between-group differences in risk–outcome associations. Existing literature on health inequity issues often discusses average differences in outcomes between social groups (Bann et al., 2020), implicitly assuming a homogeneous mechanism between groups linking risk factors throughout the range of the targeted outcome. Consequently, the findings often lead at most to the proposal of generic interventions. Instead, quantile regression-based decomposition empirically reveals both homogenous and heterogenous mechanisms operating differently between groups and within specific quantiles of the targeted outcome. As we argue shortly, this approach would enable the tailoring of intervention strategies to close between-group gaps across the outcome range.

The purpose of this study is to propose the novel use of quantile regression-based decomposition analysis to expand the empirical discussion on population health intervention strategies for health equity—namely universalism, proportionate universalism, and targeted approaches (Benach et al., 2011, 2013; Frohlich & Potvin, 2008; Marmot, 2010)—with consideration of the distributional inequalities in population health.

We follow the argument presented by Bann et al. (Bann et al., 2020), asserting that the understanding of distributional effects is consistently interpreted alongside the population strategies for public health because interventions on risk factors universally applicable to an entire population can shift the entire distribution of those risk factors (Rose, 1985). We further propose that the varying intensity of the predictor contribution across the outcome distribution support the concept of proportionate universalism (Marmot, 2010). Furthermore, the differential effect of risk factors operating between subgroups would suggest heterogeneity in the health outcome mechanisms. This would necessitate differential interventions targeting specific subgroups to effectively close the health disparity gap (Frohlich & Potvin, 2008; McLaren et al., 2010).

In the following section, we provide detailed theoretical consideration linking the distributional homogeneity/heterogeneity of health within a population to population health strategies. We then present an empirical case applying quantile regression-based decomposition results to inform interventional strategy choices. We will also discuss the potential usefulness of the proposed empirical scheme in epidemiological studies of health inequality, with a closer focus on the distributional structure of population health and its causes.

## 2 Theoretical consideration linking the distribution of health and population health strategies

The focus on the entire population is rooted in the population approach proposed by Rose (Rose, 1985) in 1985. In contrast to the high-risk approach, Rose argued that the population approach maximises the preventive effect by reducing risks across the entire population, thereby shifting the entire distribution of risks toward a lower mean. Frohlich and Potvin (Frohlich & Potvin, 2008) counterargued that the population approach may increase health inequalities if it disproportionately benefits those at a lower risk, potentially leaving behind subpopulations with certain social characteristics that put them at higher risk. Such a subpopulation requires a specific targeted intervention using the ‘vulnerable population’ approach (hereafter referred to as the targeted approach) to counter fundamental causes as proposed by Phelan et al. (Phelan et al., 2010). To leverage the advantages of universalism while addressing the differential distribution of risk factors, Marmot (Marmot, 2010) proposed ‘proportionate universalism,’ which necessitates universal actions with a scale and intensity proportional to the level of disadvantage (Francis-Oliviero et al., 2020). The concept proposed by Marmot (Marmot, 2004) is rooted in viewing health inequality as a problem for the entire population, characterised by a gradient of health status and enabling resources. Finally, Benach et al. (Benach et al., 2011, 2013) proposed a typology of health policies focusing on the potential interventional impact on the shift (mean) and shape (variance) of health status. This approach aims to effectively choose or combine targeting, universalism with targeting, redistributive, and proportionate universalism strategies (Benach et al., 2011, 2013).

When reviewing the existing debates over population health interventions, it is important to recognise that these arguments are implicitly based on different assumptions about population homogeneity. The high-risk approach, population approach in its original form, and proportionate universalism assume that people are homogeneous in their risk–outcome response characteristics but have different levels of biological, environmental, and behavioural health risks that result in the distribution of health outcomes. By contrast, the targeted approach assumes population heterogeneity, viewing populations as socially defined groups with special needs.

We argue that population homogeneity and heterogeneity are not mutually exclusive, but rather coexisting characteristics. The quantile regression-based decomposition is useful for distinguishing the degree of contribution between population homogeneity and heterogeneity. This method decomposes the outcome gap between the two groups into the ‘covariates part’ and the ‘coefficients part’. Theoretically, the ‘covariates part’ corresponds to the between-group difference explained by different levels of risk factors under the same risk factor–outcome associations. The ‘coefficients part’ corresponds to the between-group difference explained by different risk factor–outcome associations that arise from heterogeneous group characteristics. Therefore, the ‘covariates part’ demonstrates the need for proportionate universalism proposed by Marmot (Marmot, 2010), while the ‘coefficients part’ reflects the need for the targeted approach described by Frohlich and Potvin (Frohlich & Potvin, 2008). The contribution of the ‘covariates part’ and ‘coefficients part’ across the outcome distribution represents the optimal degree of combination between proportionate universalism and the targeted population approach across the outcome distribution.

## 3 Materials and methods

We will demonstrate the application of the above theorisation with a focus on the education-related disparity in the dietary behaviour of fruit and vegetable consumption, which is well known to vary clearly across educational attainment strata (Darmon & Drewnowski, 2008; Giskes et al., 2010).

Data were derived from a population-based survey conducted in metropolitan cities in Japan among residents aged 25–50 years since 2010, as described in Supplementary Data 1 (Murakami et al., 2023; Takada et al., 2014). Of the 2948 participants with valid responses (response rate: 68.7%), we excluded those who did not complete the dietary habits questionnaire (n = 141), those with missing data (n = 624; mainly for income data), and outliers for energy intake and fruit and vegetable intake (n = 12). For demonstration purposes, we limited our analysis to male participants in the lowest and highest categories of educational attainment, as they have distinct distributions of the outcome. We defined low education as high school or lower (n = 249) and high education as university or higher (n = 575).

For the outcome, dietary habits regarding fruit and vegetable intake during the preceding month were assessed using a brief-type self-administered diet history questionnaire with the energy-adjusted density method, which was validated against dietary records (Kobayashi et al., 2011). Although the food intake estimates may be susceptible to measurement errors due to misreporting, previous studies have not shown consistent reporting bias based on socioeconomic status (Grech et al., 2021).

For covariate selection, we relied on Bourdieu's habitus theory (Abel & Frohlich, 2012) and Mackenbach's discussions (Mackenbach, 2012). Education-related disparity in dietary behaviours is known to be explained by material, psychological, and social processes. The formation of the habitus depends on the availability of different forms of capitals, so we used three main types of capital (i.e., economic, social, and cultural) in this study. Economic capital, which can be in the form of money and material assets, was measured by total annual household income. Social capital, indicating material and non-material resources mobilised through inter-individual relationships, was measured by social network size (i.e., the total number of close ties (Murakami et al., 2019)) and the degree of social support (i.e., the score of perceived support by a spouse/partner or other co-residing family members (Murakami et al., 2019, Murakami et al., 2023)). Cultural capital, representing people's symbolic and informational resources for action, was measured by health literacy using the Communicative and Critical Health Literacy scale (Ishikawa et al., 2008). The details of these measurements are described in Supplementary Data 2.

We conducted the quantile regression-based decomposition proposed by Machado and Mata (Machado & Mata, 2005) and Chernozhukov et al. (Chernozhukov et al., 2013). The formula for this decomposition is presented in Supplementary Data 3. As it indicates, the ‘covariates part’ represents the between-group disparity explained by a counterfactual outcome difference for the less educated if they had the same covariate profiles as the highly educated, given the same outcome–covariate coefficients as the high education group. By contrast, the ‘coefficients part’ comprises a counterfactual outcome difference for the less educated if they had the outcome–covariate coefficients of the highly educated, given their own level of risk profiles.

All analyses were performed using Stata 16 (StataCorp LP, College Station, TX, USA). We used the command ‘sqreg’ for quantile regression and ‘cdeco’ for quantile regression-based decomposition. The bootstrapped method was applied to construct asymptotically valid confidence intervals in the quantile regression by performing multiple resampling from the original data and computing the statistics of each sample (Galvao et al., 2024). The Stata code is presented in Supplementary Data 4.

## 4 Results

**Fig. 1:**
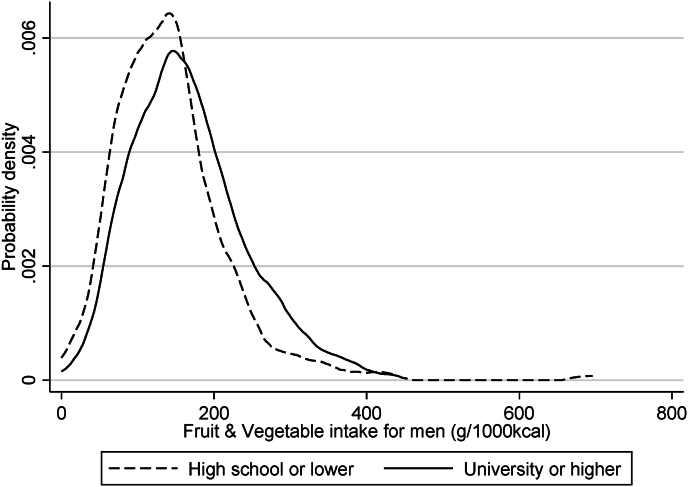
Kernel density distribution of fruit and vegetable intake by educational attainment for men (*n* = 824).

The highly educated people consumed more fruits and vegetables on average than the less educated people, although large disparities existed within the education groups (Fig. 1). Table 1 shows the results of quantile regression before stratification by education. The coefficients of educational attainment were larger in higher intake quantiles, suggesting a widening education-related gap in the higher quantile segments. Additionally, the coefficients of the covariates varied across the outcome quantiles, indicating that covariate–outcome associations differed across the outcome range. Thus, the quantile regression-based decomposition is suitable for obtaining a comprehensive understanding of risk profiles related to educational disparities.

**Table 1:** Associations of fruit and vegetable intake with education and capital variables using quantile regression (*n* = 824).

	Coefficient				
	(Lower and upper 95% confidence intervals)				
	P value				
	Q10	Q25	Q50	Q75	Q90
Education	12.3	16.4	21.1	21.1	23.0
(ref: low)	(−0.2, 24.9)	(5.9, 26.9)	(9.6, 32.7)	(6.2, 36.0)	(−6.0, 52.0)
	*0.06*	*0.002*	*<0.001*	*0.01*	*0.12*
Household	0.03	0.02	0.01	0.04	0.03
income	(0.01, 0.04)	(0.004, 0.03)	(−0.01, 0.03)	(0.02, 0.07)	(−0.003, 0.07)
	*<0.001*	*0.01*	*0.18*	*0.01*	*0.07*
Social	0.5	0.9	0.4	0.3	0.4
network	(−0.2, 1.2)	(0.4, 1.4)	(−0.1, 1.0)	(−0.5, 1.1)	(−0.9, 1.7)
	*0.18*	*<0.001*	*0.09*	*0.46*	*0.53*
Social	4.6	4.5	2.1	0.2	−4.0
support	(1.1, 8.2)	(1.4, 7.7)	(−1.5, 5.7)	(−4.0, 4.5)	(−12.3, 4.3)
	*0.01*	*0.01*	*0.25*	*0.92*	*0.34*
Health	3.4	4.9	5.6	5.4	12.7
literacy	(−6.3, 13.0)	(−3.4, 13.3)	(−3.9, 15.1)	(−8.1, 18.8)	(−8.2, 33.7)
	*0.50*	*0.24*	*0.25*	*0.43*	*0.23*
Constant	9.1	35.9	91.6	130.9	175.7
	(−33.8, 52.0)	(6.5, 65.3)	(54.1, 129.0)	(74.2, 187.7)	(104.5, 246.9)
	*0.68*	*0.02*	*<0.001*	*<0.001*	*<0.001*

Energy-adjusted values of fruit and vegetable intake were used (amount per 1000 kcal). Bootstrap replications were performed 100 times to obtain estimates of the coefficients and the 95% confidence intervals.

Table 2 presents the results of the quantile regression-based decomposition. Based on the quantile regression stratified by education groups (Supplementary Table S1 in Supplementary Data 5), the disparity in the predicted amount of fruit and vegetable intake between education groups ranged from 13.4 at the 10th percentile of the outcome intake to 41.4 at the 90th percentile. The estimated disparity was decomposed into the relative attributions of the ‘covariates part’ and ‘coefficients part’. Over the outcome range, the ‘coefficients part’ explained the major proportion of the disparity, whereas the ‘covariates part’ substantially explained approximately one-third of the group disparity in the lowest and highest quantiles. These decomposition results indicate that the two education groups were heterogeneous in their risk profiles and predominantly require group-specific ‘targeted approaches’ to close the gap. However, some common ‘proportionate universal’ strategies, with varying intensity, could be applied to the lowest and highest quantile segments as a supplemental intervention.

**Table 2:** Quantile regression-based decomposition of educational differences in fruit and vegetable intake.

	Quantile effect				
	(Lower and upper pointwise 95% confidence intervals)				
	Q10	Q25	Q50	Q75	Q90
**Predicted intake**					
University or higher	78.7	112.4	158.4	208.9	269.7
High school or lower	65.3	91.1	134.6	174.5	228.3
Difference	13.4	21.3	23.8	34.4	41.4
	(3.3, 23.5)	(10.0, 32.7)	(12.8, 34.8)	(18.1, 50.6)	(18.4, 64.4)
Contribution %	100%	100%	100%	100%	100%

**Covariates part**					
Total	5.3	4.2	5.0	8.7	15.1
	(1.6, 9.0)	(−1.0, 9.4)	(−0.5, 10.4)	(2.0, 15.3)	(5.4, 24.8)
Contribution %	39.5%	19.7%	20.9%	25.2%	36.4%

**Coefficients part**					
Total	8.1	17.1	18.9	25.7	26.3
	(−2.1, 18.3)	(4.5, 29.8)	(8.1, 29.6)	(10.0, 41.5)	(3.0, 49.7)
Contribution %	60.5%	80.3%	79.1%	74.8%	63.6%

Energy-adjusted values of fruit and vegetable intake were used (amount per 1000 kcal). Bootstrap replications were performed 100 times to obtain estimates of the quantile effects and the 95% confidence intervals. Contributions (%) were calculated by dividing each point estimate of the quantile effect by the educational difference in fruit and vegetable intake and multiplying by 100.

**Fig. 2:**
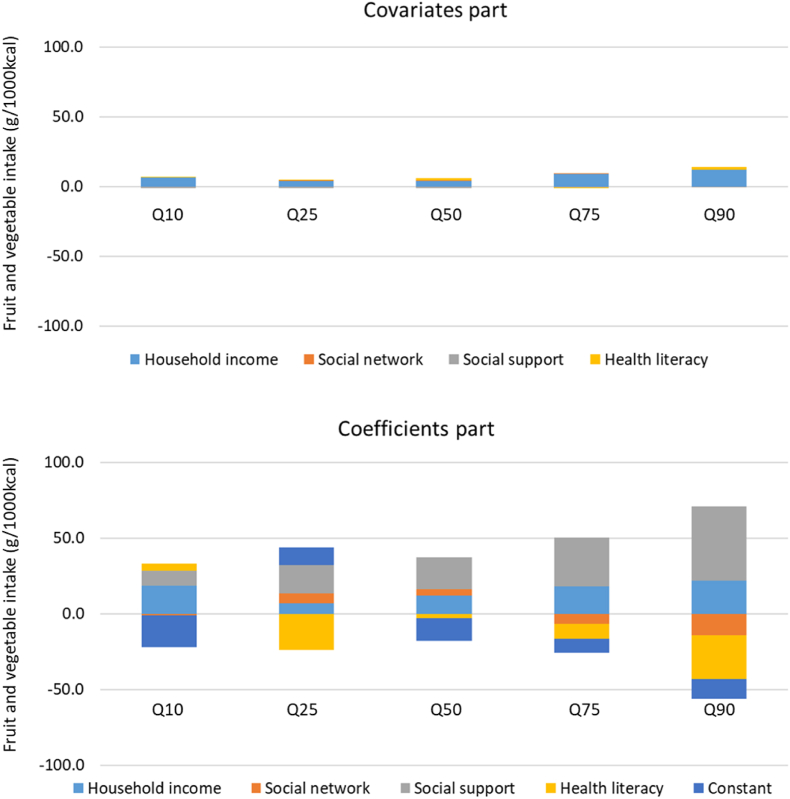
Decomposition of educational differences in fruit and vegetable intake attributable to each covariate (*n* = 824). Quantile regression-based decomposition was conducted. ‘Covariates part’ is the between-group difference explained by different levels of risk factors. ‘Coefficients part’ is the between-group difference explained by different risk factor–outcome associations.

Fig. 2 further breaks down the contributions of covariate and coefficient components for each covariate. The upper figure shows the contributions of the ‘covariates part’, indicating that household income was the main contributor across the entire outcome distribution, although its overall impact was limited. This suggests that interventions providing compensation proportional to income levels could be a potential strategy for partially closing the gap.

The lower figure shows the contributions of the ‘coefficients part’, which requires careful interpretation. For example, the coefficient contribution of income level is positive, suggesting that if the less educated had the same level of income–outcome response as the highly educated, the expected gap would close. However, the reverse is true: if the less educated had more income given their current income–outcome response, it might widen the gap. This is presumably because the highly educated are more responsive to income support, which may negate the covariate contribution of income compensation for the less educated.

Another caution is necessary regarding the coefficient contribution of social support from close ties in the higher quantiles, which may widen the gap if the less educated receive more support given their support–outcome response. This could be due to differences in the nature of personal networks between education groups, with the less educated being more vulnerable to network homophily, where close ties might reinforce less healthy dietary behaviours (Valente & Pitts, 2017). Thus, interventions aimed at changing the nature of social support would be required to effectively close the gap.

10.13039/100018696Health literacy has already contributed to closing the gap, particularly in the higher quantile segment, suggesting that providing support to help less educated individuals attain higher health literacy could promote equity by enhancing their skills to effectively find and utilize health information (Ishikawa et al., 2008). Finally, for the lowest quantile, the major driver in closing the gap was the regression constant. This suggests that unmeasured factors may be at play in explaining the disparity between groups.

## 5 Discussion

Benach et al. (Benach et al., 2011), who proposed a typology of population approaches, argued that ‘targeted and universal strategies are not mutually exclusive but complementary and build on each other’. The present study empirically supports this argument by demonstrating that quantile regression-based decomposition provides an empirical basis for designing a plausible combination of different intervention strategies for population health (Frohlich & Potvin, 2008; Marmot, 2010). Given that 60%–80% of the disparity was explained by the ‘coefficients part’ across the outcome distributions, interventions targeting specific subpopulations would be the major strategy for closing an education-related gap in the demonstrated population. Meanwhile, ‘proportionate universalism’ could also reduce the disparity in the extreme outcome quantile segments, albeit to a limited degree.

In designing a targeted approach based on the ‘coefficients part’ contribution, it is important to carefully select the intervention leverage. A positive coefficient contribution indicates that simply increasing the covariate level might widen the gap, while modifying the covariate–outcome response has the opposite effect, which can help close the gap. This aligns with the discussion on the strategic choice of interventions targeting agency (behavioural change among individuals) versus structure (conditions in which behaviour occurs) (McLaren et al., 2010).

In our demonstration, the coefficient contributions of income and family social support indicate that a targeted approach increasing these capitals among less educated people may widen the disparity, suggesting that it may counteract the covariate contributions of income, which reflect the gap-closing effect of proportionate universalism. Previous studies have pointed out that population strategies may have countervailing impacts on health inequalities (Benach et al., 2011, 2013; Frohlich & Potvin, 2008; McLaren et al., 2010), and this study has demonstrated this by quantifying the direction and extent of contributions from the ‘covariates part’ versus the ‘coefficients part’. Instead, a targeted approach involving structural modifications would be more equitable, altering the efficacy of economic resources and the nature of social support in facilitating healthy dietary choices among the less educated. For example, pricing policies (tax/subsidy) and food environment policies targeting the availability of foods in retail and food service establishments, product reformulation, and portion size adjustment could help change purchasing and intake behaviours (Hansen et al., 2022). Furthermore, given that health literacy has already shown effectiveness in closing the gap, improving health literacy at the individual level may serves as an effective leverage for further closing the gap, especially in the higher outcome quantile segments. Because health literacy is recognised as a modifiable mediator between educational attainment and dietary behaviour (Murakami et al., 2023; Stormacq et al., 2019), educational interventions, such as improving the understanding and use of nutrition labels, could be beneficial (Moore et al., 2018).

The decomposition by quantiles further indicates that the subpopulation with the lowest quantiles of fruit and vegetable intake was unique because the education-related disparity was small, while their intake remained similarly low, regardless of educational attainment. This suggests that this segment may share common risk factors that lead to low intake behaviours. The quantile regression results in Table 1 show that the lower quantile segments had a positive contribution from income and family social support, indicating that these segments might benefit from targeted interventions aimed at enhancing economic and social capital to improve their intake behaviours irrespective of educational background. Notably, this subgroup would have been overlooked if we had simply focused on the average intake of each educational group. This underscores the importance of analysing the entire distribution of health behaviours.

The proposed framework could be applicable to broader health equity issues. Population health strategies are known to carry potential risks of widening health disparities, so avoiding these unintended consequences is crucial. Previous literature examined these phenomena mainly through theoretical and simulation analysis (Benach et al., 2011, 2013; Frohlich & Potvin, 2008; McLaren et al., 2010). This study contributes to the empirical analysis of the impacts of population health strategies within the population of interest in a comprehensive and theoretically explainable manner.

Although we believe the proposed framework is promising, we must acknowledge its current limitations and the need for further refinement. First and foremost, the quantile regression and decomposition techniques do not reveal causal relationships between the covariates and outcomes. Therefore, the selection of covariates should be based on existing knowledge of the causal impact of selected risk factors. Similarly, the specification of the regression model needs to be carefully considered alongside other supplementary analyses. In our demonstration case, potential reverse causality may exist between long-term dietary habits and educational attainment. Future studies will need to address these causality issues within the context of quantile regression-based decomposition.

Second, in our demonstration, the largest contribution in the lowest quantile was the regression constant, which could be explained differently with unmeasured confounders. Technically, the bootstrap standard errors of coefficients in the quantile regression were larger at higher quantiles, leading to larger estimation errors in the decomposition results at these higher quantiles.

Third, the current form of decomposition relies on theoretically selected comparators (e.g., education) and may not fully identify the most discriminating risk factors across the outcome distribution. Machine learning methods may complement model selection and the identification of discriminating risk factors because they estimate data-driven heterogeneity in risk factors by accounting for higher-order interactions without prior assumptions (Hu et al., 2021; Subhan et al., 2023). Although hybrid approaches combining machine learning and quantile regression have been recently proposed (Hu et al., 2021; Subhan et al., 2023), there is a research gap in linking these results to population health strategies. This study suggests that the concurrent use of these methods may contribute to a better understanding of population heterogeneity and the selection of health strategies for health equity.

## 6 Conclusion

As we demonstrated, the use of quantile regression-based decomposition methods to articulate the between-group disparities in health outcomes provides an empirical basis for effectively designing population health interventions. The proposed framework explains when and how ‘targeted approaches’ and ‘proportionate universalism’ are expected to succeed or fail in promoting population health equity. This can serve as a promising tool for linking existing knowledge on the determinants of population health to the design of population health strategies. Future studies are warranted to utilise this framework and test the proposed intervention strategies in diverse settings.

## CRediT authorship contribution statement

**Ayumi Hashimoto:** Writing – original draft, Software, Methodology, Investigation, Formal analysis, Data curation, Conceptualization. **Hideki Hashimoto:** Writing – review & editing, Supervision, Methodology, Investigation, Funding acquisition, Conceptualization.

## Ethical approval

The survey procedure of the Japanese Study on Stratification, Health, Income, and Neighborhood (J-SHINE) was approved by the Research Ethics Committee of The University of Tokyo, Graduate School of Medicine (No. 3073). Participation in this study was voluntary, and written consent was obtained from each respondent. The J-SHINE Data Management Committee approved the authors’ secondary use of the data, with personally identifiable information deleted to ensure confidentiality.

## Funding

This work was supported by a Grant-in-Aid for Scientific Research on Innovative Areas [grant number 21119002] from the 10.13039/501100001700Ministry of Education, Culture, Sports, Science and Technology, Japan; by a research grant from the 10.13039/501100003478Ministry of Health, Labour and Welfare, Japan [grant number H27-Lifestyle-ippan-002]; and partially by the 10.13039/100009619Japan Agency for Medical Research and Development [grant number 24rea522101h0003]. The funders played no role in the study design; collection, analysis, or interpretation of the data; preparation, review, or approval of the manuscript; or decision to submit the manuscript for publication.

## Declaration of interests

None declared.

## Data Availability

The data will be shared on reasonable request to the J-SHINE Data Management Committee.
