# Supplementary material for: Empirical design of population health strategies accounting for the distribution of population health risks
Source: SSM Popul Health. 2024 Dec 17;29:101741. doi: 10.1016/j.ssmph.2024.101741 (PMC11729676; doi:10.1016/j.ssmph.2024.101741)
Supplement: Multimedia component 1 [file mmc1.docx]

**Supplementary Data 1. The Japanese Study on Stratification, Health, Income, and Neighborhood (J-SHINE)**

The J-SHINE conducted its first wave in 2010 in four municipalities in and around the greater Tokyo metropolitan area of Japan. Of 13 920 adults aged 25 to 50 years who were probabilistically selected from the resident registry, 8408 were contactable. These individuals were asked to complete computer-assisted self-administered questionnaires, and 4357 provided valid responses. In 2012, the second wave of the survey was conducted for participants in the first wave. Of 4294 candidates, 2948 provided valid responses (response rate: 68.7%).

Age, sex, and health literacy information was obtained from the first-wave survey, and household income, social network, social support, and dietary habit information was collected from the second-wave survey. Educational data were collected in both the first- and second- wave surveys. Some missing values for household income (*n*=664) were replaced by the annual income of the individual in single and unmarried households (*n*=18) and by the annual income of the individual and their spouse/partner in households with a spouse/partner (*n*=44). We excluded participants who did not complete the dietary habits questionnaire (*n*=141), those with missing values (*n*=624), and outliers for energy intake (<600 or >4000kcal/day; *n*=11) and fruit and vegetable intake (>1300g/1000kcal; *n*=1). Consequently, data for 2171 participants were obtained. For demonstration purposes, the analysis was limited to male participants with a high school education or lower (low education; *n*=249) and male participants with a university education or higher (high education; *n*=575).

**Supplementary Data 2. Measurement of dietary habits, income, social network, social support, and health literacy**

Dietary habits during the preceding month were assessed using a validated brief self-administered diet history questionnaire (Kobayashi et al., 2011). This questionnaire covered the consumption frequency of selected foods commonly consumed in Japan, general dietary behaviour, and usual cooking methods. The estimated intake of 58 food items and energy were calculated using a computer algorithm based on the Standard Tables of Food Composition in Japan. Energy-adjusted values of fruit and vegetable intake were calculated using the density method (amount per 1000 kcal).

Total annual household income was categorised into 15 brackets, and the median value of each category was used for analysis.

Social network size was measured by the total number of close ties (score range: ≥0) using the question, ‘How many of your colleagues/work-related persons/neighbours would you say you have close relationships with?’ (Murakami et al., 2019).

Social support was measured using the question, ‘How much do the following persons give you helpful guidance when you have a problem or are in trouble?’. Considering the influence on participants’ dietary habits, the ‘following persons’ include the individual’s spouse/partner or other co-residing family members. Two items from each source of support were rated on a 5-point scale: a lot (4), some (3), a little (2), never (1), and not applicable (1). The total score ranged from 2 to 8 points, with higher scores indicating greater perceived support (Murakami et al., 2019, 2023).

Cultural capital was measured by health literacy using the Communicative and Critical Health Literacy scale, which was developed and validated in Japan (Ishikawa et al., 2008). Five items rated on a 5-point scale were summed, divided by 5, and formed a scale score ranging from 1 to 5, with higher scores indicating greater health literacy. The Cronbach’s alpha value of the scale was 0.85.

**References for Supplementary Data 2**

Ishikawa, H., Nomura, K., Sato, M., & Yano E. (2008). Developing a measure of communicative and critical health literacy: A pilot study of Japanese office workers. *Health Promotion International*, 23, 269–74. <https://doi.org/10.1093/heapro/dan017>

Kobayashi, S., Murakami, K., Sasaki, S., Okubo, H., Hirota, N., Notsu, A., Fukui, M., & Date, C. (2011). Comparison of relative validity of food group intakes estimated by comprehensive and brief-type self-administered diet history questionnaires against 16 d dietary records in Japanese adults. *Public Health Nutrition*, 14, 1200–11. <https://doi.org/10.1017/S1368980011000504>

Murakami, K., Aida, J., & Hashimoto, H. (2019). Associations of social relationships with curative and preventive dental care use among young and middle-aged adults: Evidence from a population-based study in Japan. *Community Dentistry and Oral Epidemiology*, 47, 389–97. <https://doi.org/10.1111/cdoe.12487>

Murakami, K., Kuriyama, S., & Hashimoto H. (2023). Economic, cognitive, and social paths of education to health-related behaviors: Evidence from a population-based study in Japan. *Environmental Health Preventive Medicine*, 28, 9. <https://doi.org/10.1265/ehpm.22-00178>

**Supplementary Data 3. Quantile regression-based decomposition**

The formula for the quantile regression-based decomposition is as follows:

*Y, X*, and *j* denote outcome, covariates, and each covariate. *f* represents quantile regression, and *β* represents the coefficients. *high* and *low* indicate the high education group and low education group, respectively. In the fourth row of the equation, the counterfactual distribution is *β_j,high_X_j,low_*, derived from the conditional model of the high education group and the covariate distribution of the low education group. In the fifth row of the equation, the first term represents ‘covariates part’, or the between-group difference explained by different levels of covariates (X) between groups, given the associations of covariates with outcome in the high education group. In other words, the first term represents the counterfactual outcome difference that the low education group could have achieved if they had the same levels of covariates as the high education group given the same outcome–covariate association, which supports the idea of a proportionate universalism approach. The second term represents the ‘coefficients part’, or the between-group difference explained by different associations of covariates with outcomes between groups, given the levels of covariates in the low education group. In other words, it corresponds to the counterfactual outcome difference that the low education group could have achieved if they had similar outcome–covariate associations to those of the high education group, given their actual level of covariates. The different outcome–covariate associations indicate that the low education group had different structure of risk sets for outcomes from that in the high education group, supporting the idea of the targeted approach.

The decomposition of educational differences in fruit and vegetable intake attributable to each covariate (Figure 2) to the ‘covariates part’ and ‘coefficients part’ was calculated based on the fifth row of the equation shown above, using the estimated coefficients of quantile regression and the expected values (mean) of the covariate. In other words, contributions of the covariate (*j*) to the ‘covariates part’ and ‘coefficients part’ were and, respectively.

**Supplementary Data 4. Stata code for quantile regression and quantile regression-based decomposition**

*Run cleaning file first*

*Figure 1. Kernel density distribution of fruit and vegetable intake by educational attainment for men*

twoway kdensity adj_veg1_11_frt1_3 if edu5==1&ref_sex==1 || kdensity adj_veg1_11_frt1_3 if edu5==0&ref_sex==1, legend(order(1 "High school or lower" 2 "University or higher" )) xtitle("Fruit & Vegetable intake for men (g/1000kcal)") ytitle("Probability density")

*Table 1. Associations of fruit and vegetable intake with education and capital variables using quantile regression*

sqreg adj_veg1_11_frt1_3 edu5r h_income2012r_sc social_net_all social_support2012_12 HL_2010_5 if ref_sex==1&(edu5==1|edu5==0), quantile (.1 .25 .5 .75 .9) reps(100)

*Table 2. Quantile regression-based decomposition of educational differences in fruit and vegetable intake*

cdeco adj_veg1_11_frt1_3 h_income2012r_sc social_net_all social_support2012_12 HL_2010_5 if ref_sex==1, group(edu5) quantile (.1 .25 .5 .75 .9)

matrix list e(fitted_0)

matrix list e(fitted_1)

matrix list e(coef0)

matrix list e(coef1)

*Figure 2. Decomposition of educational differences in fruit and vegetable intake attributable to each covariate*

Excel files were used to calculate the values for Figure 2, using the estimated coefficients of quantile regression (e(coef0) and e(coef1)) and the expected values (mean) of the covariates.

*Table S1. Associations of fruit and vegetable intake with capital variables using quantile regression by education groups*

sqreg adj_veg1_11_frt1_3 h_income2012r_sc social_net_all social_support2012_12 HL_2010_5 if ref_sex==1&edu5==1, quantile (.1 .25 .5 .75 .9) reps(100)

sqreg adj_veg1_11_frt1_3 h_income2012r_sc social_net_all social_support2012_12 HL_2010_5 if ref_sex==1&edu5==0, quantile (.1 .25 .5 .75 .9) reps(100)

**Supplementary Data 5. Results of stratified quantile regression**

Table S1. Associations of fruit and vegetable intake with capital variables using quantile regression by education groups (*n*=824)

|  | High school or lower  (Low education) (*n*=249) | | | | |  | University or higher  (High education) (*n*=575) | | | | |
| --- | --- | --- | --- | --- | --- | --- | --- | --- | --- | --- | --- |
|  | Coefficient | | | | |  | Coefficient | | | | |
|  | (Lower and upper 95% confidence intervals) | | | | |  | (Lower and upper 95% confidence intervals) | | | | |
|  | *P value* | | | | |  | *P value* | | | | |
|  | Q10 | Q25 | Q50 | Q75 | Q90 |  | Q10 | Q25 | Q50 | Q75 | Q90 |
| Household | −0.004 | 0.01 | −0.001 | 0.01 | 0.02 |  | 0.03 | 0.02 | 0.02 | 0.04 | 0.06 |
| income | (−0.05, 0.04) | (−0.03, 0.05) | (−0.04, 0.04) | (−0.04, 0.06) | (−0.03, 0.07) |  | (0.01, 0.04) | (0.001, 0.03) | (0.001, 0.04) | (0.02, 0.07) | (0.02, 0.11) |
|  | *0.87* | *0.70* | *0.96* | *0.62* | *0.46* |  | *<0.001* | *0.04* | *0.04* | *<0.001* | *0.002* |
| Social | 0.4 | 0.5 | −0.01 | 1.4 | 2.1 |  | 0.3 | 0.9 | 0.5 | 0.2 | −0.3 |
| network | (−0.4, 1.1) | (−0.8, 1.8) | (−1.7, 1.7) | (−1.3, 4.1) | (−1.0, 5.1) |  | (−0.8, 1.4) | (0.5, 1.4) | (−0.2, 1.3) | (−0.6, 1.0) | (−1.2, 0.7) |
|  | *0.32* | *0.45* | *0.99* | *0.31* | *0.18* |  | *0.63* | *<0.001* | *0.13* | *0.66* | *0.56* |
| Social | 3.8 | 2.3 | 0.5 | −4.1 | −8.3 |  | 6.1 | 5.8 | 4.3 | 2.0 | 0.9 |
| support | (−2.0, 9.5) | (−2.6, 7.1) | (−6.2, 7.2) | (−14.0, 5.7) | (−21.7, 5.1) |  | (1.4, 10.8) | (1.6, 10.0) | (−1.0, 9.7) | (−3.1, 7.2) | (−11.1, 12.9) |
|  | *0.20* | *0.35* | *0.88* | *0.41* | *0.22* |  | *0.01* | *0.01* | *0.11* | *0.43* | *0.88* |
| Health | −0.2 | 9.0 | 6.9 | 2.7 | 17.4 |  | 2.2 | 3.6 | 5.7 | 1.5 | 9.4 |
| literacy | (−29.6, 29.2) | (−7.0, 25.0) | (−5.6, 19.5) | (−18.3, 23.8) | (−5.1, 39.8) |  | (−6.5, 10.9) | (−5.3, 12.5) | (−7.1, 18.5) | (−18.6, 21.5) | (−15.9, 34.7) |
|  | *0.99* | *0.27* | *0.28* | *0.80* | *0.13* |  | *0.62* | *0.43* | *0.38* | *0.89* | *0.47* |
| Constant | 47.4 | 39.4 | 106.8 | 169.6 | 181.1 |  | 18.4 | 50.7 | 94.6 | 156.7 | 168.5 |
|  | (−45.2, 140.0) | (−11.6, 90.4) | (61.3, 152.3) | (73.4, 265.8) | (86.9, 275.9) |  | (−21.9, 58.6) | (18.9, 82.6) | (54.4, 134.8) | (81.3, 232.2) | (71.2, 265.8) |
|  | *0.31* | *0.13* | *<0.001* | *0.001* | *<0.001* |  | *0.37* | *0.002* | *<0.001* | *<0.001* | *0.001* |

Energy-adjusted values of fruit and vegetable intake were used (amount per 1000 kcal). Bootstrap replications were performed 100 times to obtain estimates of the coefficients and the 95% confidence intervals.
